# Heart Injury with Projectile Lodged Inside the Heart

**DOI:** 10.21470/1678-9741-2018-0012

**Published:** 2018

**Authors:** Marco Antônio Volpe, Jorge Edwin Morocho Paredes, Luciana Costacurta Redigolo, Isaac Samuel Moscoso Sanchez, Fernando Lanichek, Luiza Zita D'Albuquerque Silveira

**Affiliations:** 1 Centro para Assistência Integral em Cardiologia (CERAIC), São Paulo, SP, Brazil.; 2 Hospital Santa Catarina, São Paulo, SP, Brazil.

**Keywords:** Heart injuries, Wounds and Injuries, Wounds, Gunshot, Cardiac Surgical Procedures

## Abstract

Penetrating heart injuries present high mortality rates. Increasing rates of urban violence have contributed to a significant rise in the number of heart injuries by firearm projectiles. Such injuries are associated with the highest mortality rates among penetrating cardiac injuries and may involve one or more cardiac chambers. We present the case of a police officer who, in an approach to five robbers, suffered a transfixed cardiac injury by firearm with the projectile having been lodged inside the right ventricle. This patient was successfully operated, 65 days after the injury, at our institution.

## INTRODUCTION

Despite advances in medicine that have high technology to support critical patients, penetrating cardiac injuries still pose a challenge to cardiothoracic surgery teams because of the high mortality rates. The high number of deaths in the prehospital phase impairs the calculation of the real mortality rates, but values are estimated between 16% to 97%^[^^1^^]^. The mechanism of trauma, the clinical conditions at hospital admission, and the presence of lesions in multiple cardiac chambers are the most important variables for the determination of the outcome in these patients^[^^1^^,^^2^^]^. The main causes of death are the hypovolemic shock due to exsanguination, and the cardiac tamponade^[^^2^^]^. Currently, heart injuries have been more common due to the rampant growth of urban violence and the easy access of the civilian population to firearms^[^^1^^,^^3^^]^.

## CASE REPORT

**Fig. 1:**
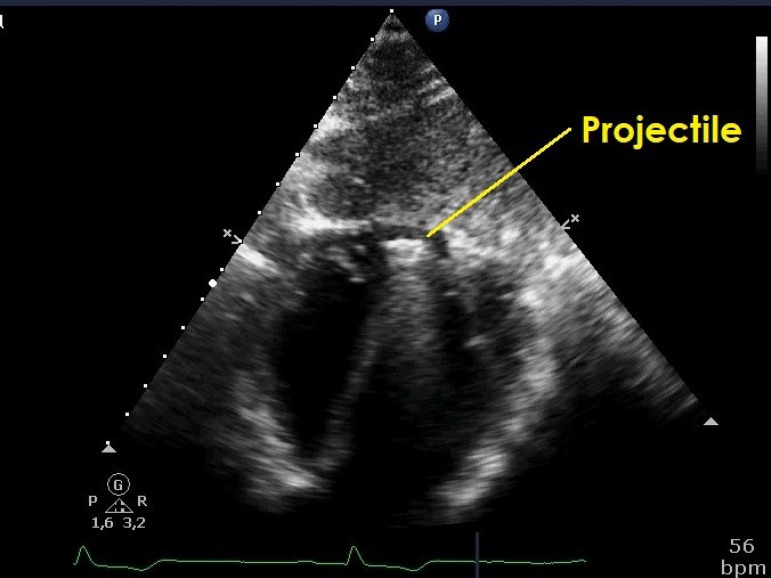
Echocardiogram showing the projectile inside the right ventricle.

**Fig. 2:**
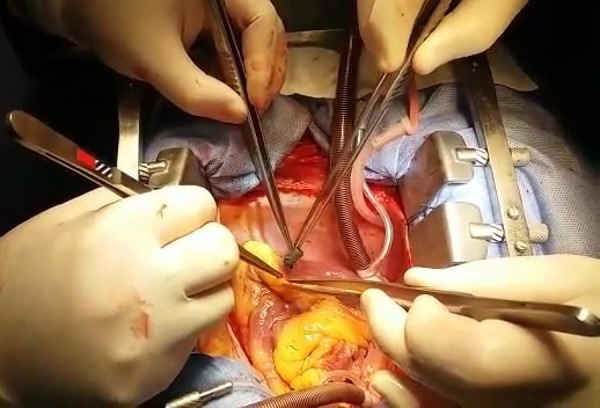
Removal of the projectile from the right ventricle.

LSA, a 34-year-old male police officer, came to the office to report that he had been discharged 16 days after being treated for chest injury by firearm. He reported that the projectiles (two) were still in his body. He reported having been treated in the public hospital emergency room with two projectiles orifices entries in the lateral side (subaxillary), on the average height of the right hemithorax, provoked during the approach of robbers in an attempt of assault. According to a copy of the admission record, he was slightly discolored, peripheral perfusion maintained, tachycardic, and with decreased vesicular murmur on the right chest. Initial stabilization support measures were established. Chest X-rays showed one projectile in anterior cardiac topography, near the apex of the heart and another in the right rectus abdominis, near the thoracoabdominal transition. In addition, it showed moderate right pleural effusion. Computed tomography of the chest ratified the radiographic findings and showed discrete pericardial effusion. The right hemithorax was drained, and then the patient was transferred to a private hospital, where the radiographs and tomography of the chest were repeated, besides transthoracic and transesophageal echocardiograms. None of them report the presence of a projectile within the heart. All reports referred to the presence of a projectile in topography around the heart - nearby. During the hospitalization, the patient had a right pulmonary embolism, and was anticoagulated with rivaroxaban, being discharged after two weeks. Upon analyzing the case in detail, we could not rule out the possibility that one of the projectiles was lodged inside the heart. New imaging exams were requested from the outpatient in another institution. However, the reports remained imprecise as to the exact location of the projectile in cardiac topography. After a clinical meeting for discussion of all the exams, a new echocardiogram was elected, which, this time, showed the projectile inside the right ventricle (Figure 1). The patient was promptly hospitalized and underwent surgical intervention. Access to the thoracic cavity was made by median sternotomy. No hematoma was observed in the pericardial fat, and after the pericardiotomy, the orifice of entry of the projectile in the pericardial cavity was not found. Only discrete sero-sanguineous effusion was found. Extracorporeal circulation was established by drainage of both vena cava and infusion by the ascending aorta. A tactile inspection of the diaphragmatic wall of the right ventricle was performed under total aortic clamping and cardioplegic arrest with Custodiol(r). This maneuver allowed us to feel the presence of the projectile near the apex of this ventricle. Unbelievably, the diaphragmatic wall of the right ventricle did not show the projectile's inlet orifice. A ventriculotomy of approximately 3 cm was performed on the diaphragmatic face of the right ventricle followed by removal of the projectile (Figure 2). Right ventricle closure was performed with separate "U" points (3.0 polypropylene) anchored in two Teflon(r) bars. Extracorporeal circulation was discontinued as soon as hemodynamic conditions allowed. Through the median incision the other projectile, which had been lodged in the right rectus abdominis, was located and removed. Surgery was completed with the revision of hemostasis, mediastinal drainage, and closure of the thorax. Trans and postoperative echocardiograms did not show the presence of interventricular communication. The patient presented a good evolution when he was discharged, thus returning to his normal healthy life.

## DISCUSSION

Despite the improvement in trauma patient care, a large number of victims of penetrating cardiac injuries die before their admission to a hospital^[^^1^^]^. Most are caused by white weapons or firearm projectiles^[^^1^^,^^2^^]^, although such injuries may also be caused by foreign bodies, costal or sternal fractures^[^^1^^]^ under more rare circumstances. Penetrating cardiac injuries by white weapons are more frequent than firearms, but the increase in urban violence rates has contributed to the growing importance of the firearms injuries^[^^1^^,^^3^^]^. Degiannis et al.^[^^3^^]^, analyzing 117 patients with cardiac trauma by white weapons or firearm projectiles, showed that in the victims of firearm injuries, mortality reached 81%, being statistically significant when compared to the 15.6% associated with injuries due to white weapons. The same data were not found by Rodrigues et al.^[^^4^^]^, whom, in an analysis of 70 patients with penetrating cardiac wounds by white weapons or firearms, found no statistical differences in mortality between the two groups. As said previously, the high number of deaths in the prehospital phase impairs the calculation of the real mortality rates and may justify, at least in part, the observed differences. Degiannis et al.^[^^3^^]^ also found no differences in mortality rates when comparing wounds in one or multiple cardiac chambers. Lone et al.^[^^5^^]^ studied 40 patients considering only heart injuries by projectiles of firearms and shrapnel and showed that 87.5% had a lesion in a single chamber and that in this group survival was 62.8%. The 12.5% with lesions in more than one chamber presented 100% mortality. The present patient had a transfixing lesion that affected the right ventricle, with the projectile lodged inside this cardiac chamber. This occurrence characterizes it as a rare case, given the mechanism of the trauma, the final location of the projectile and the outcome presented. After all, it did not evolve either to death due to cardiac injury nor to the resulting pulmonary embolism. Similar to this report, is Meira et al.^[^^6^^]^ report case, in which the projectile lodged inside the right ventricle was removed 18 days after the trauma. In this particular case, the projectile was also lodged inside the right ventricle, but 65 days were elapsed between the lesion and its removal. Ventricular penetrating lesions tend to bleed less intensely than atrial ones because they are stagnant during myocardial contractions^[^^2^^,^^6^^]^, a fact that may have been relevant to the favorable outcome presented here. In this way, another relevant point may have been the location near the apex of the right ventricle, a trabecular region, which may have contributed to the containment of the bleeding.

**Table:** 

Authors' roles & responsibilities	
MAV	Substantial contributions to the conception or design of the work; or the acquisition, analysis, or interpretation of data for the work; final approval of the version to be published
JEMP	Substantial contributions to the conception or design of the work; or the acquisition, analysis, or interpretation of data for the work; final approval of the version to be published
LCR	Substantial contributions to the conception or design of the work; or the acquisition, analysis, or interpretation of data for the work; final approval of the version to be published
ISMS	Substantial contributions to the conception or design of the work; or the acquisition, analysis, or interpretation of data for the work; final approval of the version to be published
FL	Substantial contributions to the conception or design of the work; or the acquisition, analysis, or interpretation of data for the work; final approval of the version to be published
LZDAS	Substantial contributions to the conception or design of the work; or the acquisition, analysis, or interpretation of data for the work; final approval of the version to be published
